# Functional significance of protein assemblies predicted by the crystal structure of the restriction endonuclease BsaWI

**DOI:** 10.1093/nar/gkv768

**Published:** 2015-08-03

**Authors:** Gintautas Tamulaitis, Marius Rutkauskas, Mindaugas Zaremba, Saulius Grazulis, Giedre Tamulaitiene, Virginijus Siksnys

**Affiliations:** Department of Protein–DNA Interactions, Institute of Biotechnology, Vilnius University, Graiciuno 8, LT-02241 Vilnius, Lithuania

## Abstract

Type II restriction endonuclease BsaWI recognizes a degenerated sequence 5′-W/CCGGW-3′ (W stands for A or T, ‘/’ denotes the cleavage site). It belongs to a large family of restriction enzymes that contain a conserved CCGG tetranucleotide in their target sites. These enzymes are arranged as dimers or tetramers, and require binding of one, two or three DNA targets for their optimal catalytic activity. Here, we present a crystal structure and biochemical characterization of the restriction endonuclease BsaWI. BsaWI is arranged as an ‘open’ configuration dimer and binds a single DNA copy through a minor groove contacts. In the crystal primary BsaWI dimers form an indefinite linear chain via the C-terminal domain contacts implying possible higher order aggregates. We show that in solution BsaWI protein exists in a dimer-tetramer-oligomer equilibrium, but in the presence of specific DNA forms a tetramer bound to two target sites. Site-directed mutagenesis and kinetic experiments show that BsaWI is active as a tetramer and requires two target sites for optimal activity. We propose BsaWI mechanism that shares common features both with dimeric Ecl18kI/SgrAI and *bona fide* tetrameric NgoMIV/SfiI enzymes.

## INTRODUCTION

Type II restriction endonucleases recognize short 4–8 bp nucleotide sequences and cleave phosphodiester bonds within or close to their target site. They exhibit a wide range of subunit composition (monomer, dimer or tetramer), domain organization and cofactor requirements and are categorized into 11 different subtypes (1). Active sites of Type II restriction enzymes belong to four nuclease families PD-(D/E)XK, HNH, PLD and GIY-YIG (2). Despite the common function, amino acid sequences of restriction endonucleases share little sequence similarity (2). Nevertheless, structural and bioinformatics studies revealed that a group of restriction endonucleases that share CCGG motif within their target sites (termed here ‘CCGG-family’) are evolutionary related (3,4). To date, seven crystal structures of the CCGG-family endonucleases, specific for the CCGG tetranucleotide embedded into five different sequence contexts have been solved (Table 1): NgoMIV (recognition sequence 5′-G/CCGGC-3′, ‘/’ denotes the cleavage site), Cf10I and Bse634I (5′-R/CCGGY-3′ (R - purine, Y- pyrimidine)), SgrAI (5′-CR/CCGGYG-3′), Ecl18kI (5′-/CCNGG-3′), EcoRII and PspGI (5′-/CCWGG-3′ (W - A or T))(5–12). Structure comparison shows that all these enzymes have a similar fold and share structural determinants for the CCGG sequence recognition; however, they use unique structural elements for the discrimination of variable nucleotides outside the conserved CCGG core. Moreover, CCGG-family enzymes differ by domain organization, oligomeric structure, etc. (Table 1). First, they exhibit distinct domain architecture: for example, EcoRII has separate catalytic and effector domains, while the other CCGG-family enzymes are single domain proteins (13). Next, they exist in different oligomeric forms: PspGI and EcoRII are dimers, Ecl18kI and SgrAI are dimers in the apo-form and form tetramers or oligomers when bound to DNA, while other CCGG-family enzymes are tetramers. Moreover, for an optimal DNA cleavage the CCGG-family enzymes require binding of one (PspGI), two (NgoMIV, Cfr10I, Bse634I, Ecl18kI, SgrAI) or three (EcoRII) DNA targets (7,14–20). To accommodate interrupted CCGG sequences in their conserved active sites Ecl18kI, EcoRII and PspGI enzymes employ an elegant nucleotide flipping mechanism (5,9,12,21).

**Table 1.** Structurally characterized CCGG restriction endonucleases

Restriction endonuclease	Recognition sequence	Subtype	Number of sites required for optimal activity	Oligomeric state	PDB ID	References
Bse634I	R/CCGGY	IIF	2	tetramer	1KNV, 3V1Z, 3V20, 3V21	(10,11,18)
Cfr10I	R/CCGGY	IIF	2	tetramer	1CFR	(14,16)
NgoMIV	G/CCGGC	IIF	2	tetramer	1FIU, 4ABT	(7)
SgrAI	CR/CCGGYG	IIF	2	dimer/‘run-on’ oligomer	3DVO, 3DPG, 3MQY, 4C3G, 3DW9, 3N7B, 3MQ6	(8,15,48,49)
Ecl18kI	/CCNGG	IIF	2	dimer/tetramer	2FQZ	(4,5,20)
EcoRII-C	/CCWGG	IIE	3	dimer	1NA6, 3HQG	(9,13,19)
PspGI	/CCWGG	IIP	1	dimer	3BM3	(12,17)

To understand how different sequence specificities are achieved within the CCGG-family of restriction endonucleases, we have focused on structural and functional characterization of the BsaWI restriction endonuclease from the thermophilic bacterium *Bacillus stearothermophilus* W1718. BsaWI recognizes a hexanucleotide 5′-W/CCGGW-3 sequence that accommodates W (A and T) nucleotides outside the CCGG core but rejects S (G and C) nucleotides. We have solved a crystal structure of BsaWI in complex with a cognate oligoduplex. The crystal structure of the BsaWI–DNA complex reveals that the protein is able to form tetramers and higher order oligomers through multimerization of the primary dimers. We show that in solution BsaWI protein exists in a dimer-tetramer-oligomer equilibrium, but in the presence of specific DNA forms a tetramer bound to two target sites. Kinetic analysis demonstrates that BsaWI tetramer binds two cognate DNA copies and cleaves them in a concerted manner. A disruption of the tetramerization contacts identified in the crystal leads to dimeric variants with a compromised cleavage activity.

## MATERIALS AND METHODS

### Oligonucleotides

All oligonucleotides used in this study were synthesized by Metabion (Martinsried, Germany). DNA oligoduplexes and fragments used in crystallization, DNA binding and cleavage studies are presented in Supplementary Table S1.

### Cloning and mutagenesis

A plasmid bearing an R.BsaWI gene pACYCT7BsaWI.R was kindly provided by New England Biolabs. The *bsaWI.R* gene was cloned into pBAD24 expression vector using standard procedures. BsaWI mutants were obtained by the modified QuickChange Mutagenesis Protocol (22). Sequencing of the entire genes of the mutants confirmed that only the designed mutations have been introduced.

### Protein expression and purification

The wild-type (wt) and the mutant BsaWI proteins were expressed in *Escherichia coli* DH10B (ara-) cells carrying plasmids pACYC184-MspI.M [Cm^R^] and pBAD24-BsaWI.R [Ap^R^] (or a corresponding plasmid, containing the mutant *bsaWI.R* gene), were grown in the LB medium supplemented with 30 mg/ml chloramphenicol (Cm) and 50 mg/ml carbenicillin (Cb). Protein expression was performed by cultivation for 16 h at 16°C in the presence of 0.2% (w/v) arabinose. Se-Met labeled BsaWI was prepared by inhibiting the methionine synthesis pathway and expressing the protein in the presence of D,L-selenomethionine (Sigma) following the published procedure (23). For purification the cells were re-suspended in a the Purification Buffer (10 mM K-phosphate (pH 7.4), 0.2 M NaCl, 1 mM ethylenediaminetetraacetic acid (EDTA), 7 mM 2-mercaptoethanol, 5% (v/v) glycerol) supplemented with 2 mM phenylmethanesulfonylfluoride (PMSF) and sonicated. The supernatant was purified through a subsequent chromatography on phosphocellulose P11 (Whatman) and Q-Sepharose (GE Healthcare) columns by elution with 0.2–1.5 M NaCl gradients in the Purification Buffer. Fractions containing the target protein were dialyzed against the storage buffer (10 mM Tris–HCl (pH 7.4), 300 mM KCl, 1 mM DL-Dithiothreitol (DTT), 1 mM EDTA, 50% glycerol) and stored at −20°C. Concentrations of the wt, mutant and Se-Met labeled proteins were determined by measuring UV absorbance at 280 nm using appropriate extinction coefficients calculated by the ProtParam tool (http://web.expasy.org/protparam/). All protein concentrations are given in terms of dimer if not stated otherwise.

### Crystallization and structure determination

BsaWI and Se-Met labeled BsaWI were mixed with the oligoduplex SP14 (Supplementary Table S1) in ratio 1:1.2 and the complexes were concentrated to 1–1.5 mg/ml. Crystals were grown using the sitting drop vapor diffusion method at 19°C by mixing 0.5 μl of the complex solution with 0.5 μl of the crystallization buffer (0.02 M CaCl_2_, 0.1 M Na-succinate (pH 5.0), 30% (v/v) (+/−)-2-Methyl-2,4-pentanediol). Crystals belonging to the space group C222_1_ appeared after 1 day. The X-ray diffraction datasets for native and Se-Met modified protein crystals were collected at the EMBL/DESY DORIS X12 beamline (Germany) at 100 K without additional cryoprotection. MOSFLM (24), SCALA and TRUNCATE (25) were used for data processing. A native dataset was collected from a crystal soaked in 1 mM HgCl_2_ for 3 days; however Hg anomalous signal was not present and therefore we treated it as ‘native’. Data collection and refinement statistics is presented in the Table 2.

**Table 2.** Data collection and refinement statistics

	Native	Se-Met
*Data collection statistics*		
Space group	C222_1_	C222_1_
A (Å)	89.562	90.430
B (Å)	136.336	137.803
C (Å)	73.482	73.057
Wavelength	0.9887	0.9762
X-ray source	X12	X12
Total reflections	600277	185223
Unique reflections	41718	12901
Resolution range (Å)	29.16–1.80	32.27–2.7
Completeness (%) (last shell)	99.4 (95.9)	99.9 (100.0)
Multiplicity (last shell)	14.4 (12.1)	14.4 (13.3)
I/σ (last shell)	6.3 (1.4)	6.7(4.1)
R(merge) (%) (last shell)	6.7 (54.1)	7.9 (18.0)
B(iso) from Wilson (Å^2^)	26.3	48.8

*Refinement statistics*		
Resolution range (Å)	28.4–1.8	
Reflections work/test	80028 (41703 non-anomalous)/8024	
Protein atoms	2298	
DNA atoms	284	
Hetero atoms (SIN, Ca)	18	
Solvent molecules	247	
R-factor (%)	0.1524	
R-free (%)	0.1879	
R.m.s.d. bond lengths (Å)	0.019	
R.m.s.d. angles (deg)	1.659	
Ramachandran core region (%)	97.19	
Ramachandran allowed region (%)	2.81	
Ramachandran disallowed region (%)	0	

The structure was solved using the Se-Met modified protein by the SIRAS protocol of Auto-Rickshaw (26). Sixteen heavy atoms were found using the programs SHELXCD (27,28). The correct hand for the substructure was determined using the programs ABS (29) and SHELXE (30). The occupancy of all substructure atoms was refined using the program BP3 (31). The initial phases were improved using density modification and phase extension to 2.70 Å resolution using the program RESOLVE (32). A partial alpha-helical model was produced using the program HELICAP (33). The partial model contained 344 residues out of the total number of 272 residues (protein was modeled also in place of the DNA). The partial model was used in molecular replacement in native dataset at 2.40 Å resolution by the program MOLREP (34), following refinement with REFMAC5 (35) and automated model-building with ARP-wARP (33). The new partial model contained 278 residues in 3 chains, a total of 257 residues have been docked. Manual model rebuilding and DNA placement was performed in COOT (36). The structure was refined with REFMAC5 and phenix.refine.1.8.3 (37). Translation/Libration/Screw-motion (TLS) groups were determined by TLSMD server (http://skuld.bmsc.washington.edu/∼tlsmd/) (38).

The contact surfaces buried between the two molecules were calculated using PISA server (http://www.ebi.ac.uk/pdbe/prot_int/pistart.html) (39). Protein–DNA contacts were analyzed by NUCPLOT (40). All molecular scale representations were prepared using MOLSCRIPT (41) and RASTER3D (42) software.

### Gel filtration

Gel filtration was carried out at room temperature on an ÄKTA FPLC system (GE Healthcare) using a Superdex 10/200 HR column (Amersham Pharmacia Biotech) pre-equilibrated with 20 mM Tris–HCl (pH 7.4), 0.5 M NaCl and 5% (v/v) glycerol. Protein samples at 0.25–25 μM loading concentration were prepared in 70 μl of the above buffer. Elution from the column was monitored by measuring absorbance at 280 nm. The apparent molecular weights of proteins were evaluated from the elution volume using a series of standards (Gel filtration Calibration Kit from GE Healthcare).

### Gel mobility shift assay

DNA binding by wt BsaWI and the mutants was analyzed by the electrophoretic mobility shift assay (EMSA) using a 30-bp cognate SP or 33-bp non-cognate NSP ^33^P-labeled DNA duplexes (Supplementary Table S1). DNA (final concentration 1 nM) was incubated with wt or mutant BsaWI (final concentrations of proteins varied from 0.02 to 1000 nM) for 15 min in 20 μl of 40 mM Tris–acetate (pH 8.3), 0.1 mg/ml bovine serum albumin (BSA) and 10% glycerol containing either 0.1 mM EDTA or 5 mM CaCl_2_ at room temperature. Free DNA and protein–DNA complexes were separated by electrophoresis using 8% acrylamide gels (29:1 acrylamide/bisacrylamide in 40 mM Tris-acetate (pH 8.3), and 0.1 mM EDTA or 5 mM CaCl_2_). Electrophoresis was run at room temperature for 2–3 h at 6 V/cm. Radiolabeled DNA was detected and quantified using the Cyclone phosphorimager and the OptiQuant software (Packard Instrument). The dissociation constant values were calculated as described in (43).

### DNA cleavage assay

DNA fragments containing one- or two-sites (termed 1-site and 2-site DNAs, 406 and 630 bp of length, respectively, see Supplementary Table S1) were obtained performing polymerase chain reaction with pACYC184 plasmid and corresponding primers. Cleavage reactions were performed at 37°C in the Reaction Buffer (33 mM Tris–acetate (pH 7.9), 10 mM Mg-acetate, 66 mM K-acetate, 0.1 mg/ml BSA buffer). For the 1-site and 2-site DNA cleavage, the reactions contained 10 nM of the 5′-^33^P labeled DNA fragments and 20–7000 nM of protein. Activation of the 1-site DNA fragment cleavage by SP and NSP oligoduplexes (Supplementary Table S1) was performed at 300 nM protein concentration and varying SP/NSP concentration at 30–6000 nM. Aliquots were removed at timed intervals and quenched with the loading dye solution (75 mM EDTA (pH 8.0), 0.025% bromphenol blue, 0.3% sodium dodecyl sulphate and 50% (v/v) glycerol). Non-denaturing 8% acrylamide gels (29:1 acrylamide/bisacrylamide in 40 mM Tris-acetate (pH 8.3)) were run at 12 V/cm. A 30-bp length ^32^P labeled hairpin DNA oligoduplex HP (Supplementary Table S1) was used to test the strand cleavage preference. The final concentration of DNA in the reaction mixture was 10 nM, concentration of protein was 15–1000 nM. Aliquots were removed at timed intervals and quenched with the loading dye solution (1 M formamide, 50 mM EDTA, 0.025% bromophenol blue). Denaturing 20% acrylamide gels (29:1 acrylamide/bisacrylamide in 45 mM Tris-borate (pH 8.3), 6 M urea, 1 mM EDTA buffer) were run at 36 V/cm. The radiolabeled DNA was detected and quantified as described above. The DNA cleavage rate constants were determined by fitting single exponentials to the substrate depletion data. Non-linear regression analysis employed the KYPLOT 2.0 software (44).

## RESULTS

### Crystal structure of BsaWI

**Figure 1.**
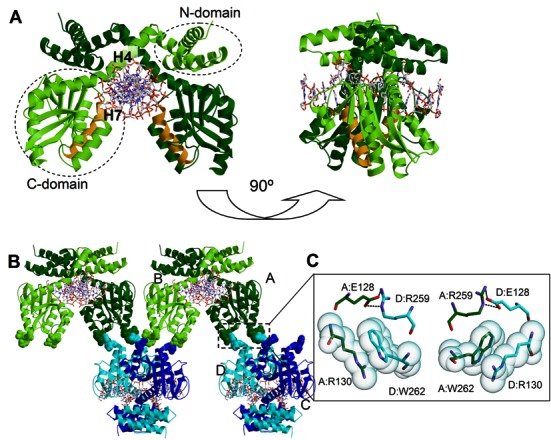
The crystal structure of the BsaWI–DNA complex. (**A**) BsaWI dimer. The BsaWI subunits are colored light green and dark green, respectively. Domains are shown by dotted circles. Helix H4 connects the C-domain to the N-domain. Helix H7 (orange) corresponds to the dimerization helices of Bse634I, Cfr10I, NgoMIV. (**B**) BsaWI oligomers. The BsaWI dimers (the primary dimers are colored green and blue) form a network in the crystal. The primary dimers AB and CD form a putative tetramer. The interacting residues are depicted in the space-fill mode. One of the putative tetramerization/oligomerization interfaces is boxed. (**C**) The putative tetramerization/oligomerization interface of BsaWI. In the closeup view the residues involved in the tetramerization contacts are shown and colored according to the subunit color.

**Figure 2.**
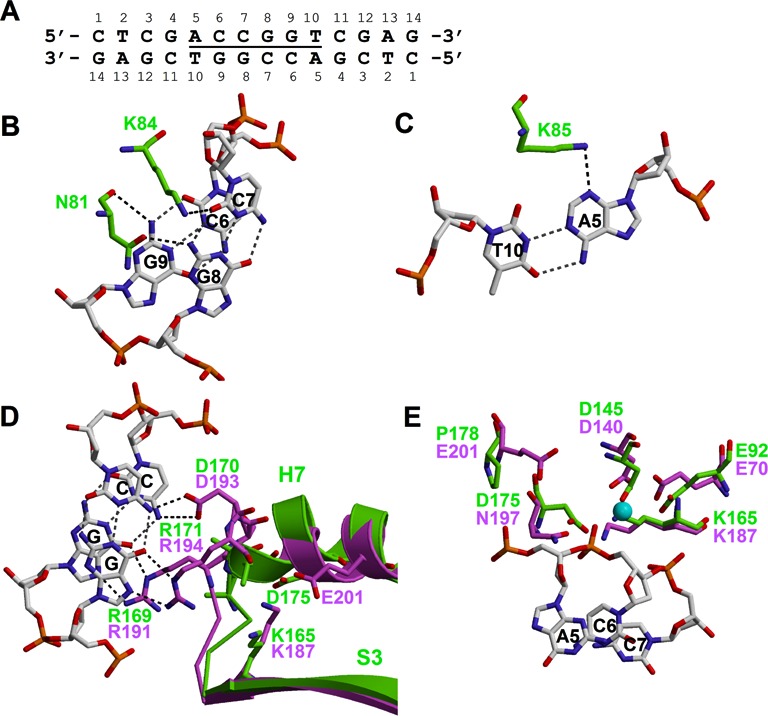
BsaWI–DNA contacts. (**A**) DNA oligoduplex used in crystallization. (**B**) BsaWI contacts with the CCGG tetranucleotide from the minor groove side. (**C**) Recognition of the outer base pair by BsaWI. (**D**) The NgoMIV R-(D/E)R residues (violet, 4ABT) overlaid with the BsaWI putative CCGG recognition residues (green). Structural elements of NgoAVII and BsaWI (S3 and H7) bearing the catalytic lysine and glutamate/aspartate residues are also shown. DNA is from the NgoMIV complex. (**E**) The active site residues of BsaWI (green) overlaid with the active site of NgoMIV (violet, PDB ID: 4ABT). DNA and Ca^2+^ ion (cyan sphere) from the NgoMIV-DNA structure are shown.

We have purified the BsaWI protein from heterologous *E. coli* strain and solved the crystal structure in complex with 14-bp oligoduplex SP14 containing one variant of the BsaWI recognition sequence 5′-ACCGGT-3′ (Figures 1 and 2A). The asymmetric unit contains one protein and one DNA chain; a dimer can be generated by rotating protein–DNA chains by the crystallographic two-fold axis.

The BsaWI protein is folded into two domains: the N-terminal helical domain composed of α helices H1-H3 (residues 1–69; termed N-domain) and the C-terminal catalytic domain (residues 70–272; termed C-domain) (Figure 1A, Supplementary Figure S1). The C-domain is folded into a six-stranded β sheet flanked by α helices H4-H9. The domains are connected by a long α helix H4.

DALI structure similarity search revealed that BsaWI is most similar to the CCGG-family restriction endonucleases NgoMIV (PDB ID 4ABT, Z-score 9.3), Bse634I (3V21, 8.9), Cfr10I (1CFR, 8.8) and SgrAI (3MQ6, 8.4) (45) that recognize hexanucleotide or octanucleotide sequences.

#### Dimer structure

BsaWI forms a stable dimer by swapping the N-domains, while the C-domains are located apart of each other (Figure 1A). The N-domains make extensive contacts with the C-domains of the other protein subunit. The dimer interface is large (∼2000 Å^2^) and involves both hydrogen bonds and hydrophobic interactions. Helix H7 of the C-domain corresponds to the recognition/dimerization helices of Bse634I, Cfr10I, NgoMIV and other related restriction endonucleases; however in the crystal structure it is not involved in the dimerization contacts (Figure 1A, Supplementary Figure S1).

#### Tetramerization/oligomerization interface

In the crystal structure, the BsaWI primary dimers form a linear chain of protein molecules via C-domain contacts (Figure 1B). To explore whether these contacts are important for the BsaWI activity, we examined the putative oligomerization interface of BsaWI. Three motifs located on the N-terminal part of the helix H6 and C-termini of the helices H7 (bearing catalytic D175 and the R_169_D_170_R_171_ motif) and H9 from each BsaWI subunit are involved in the presumed tetramerization/oligomerization contacts (Figure 1B). In the putative tetramerization/oligomerization interface E128 from one subunit makes a salt bridge with R259 from the other subunit and R130 makes a cation–π interaction with the aromatic ring of W262 (Figure 1C). The contact interface area is 514 Å^2^ and PISA server (39) scores it as unimportant for the complex formation. However, this contact interface coincides with the tetramerization interface of the NgoMIV, Bse634I and Ecl18kI suggesting that it might be important for the activity of BsaWI (5,7,10).

#### Contacts with DNA

In the crystal BsaWI forms a dimer in an ‘open’ configuration that approaches the DNA molecule from the minor groove side and makes no sequence specific contacts to DNA bases in the major groove; therefore we term this complex a pre-specific complex (Figure 1A). In the pre-specific complex BsaWI makes extensive contacts to the DNA backbone. Direct and water-mediated contacts to seven backbone phosphates of each DNA strand are made by eleven residues from each subunit, mostly from the N-domain and from the H4 helix.

In the crystal BsaWI contacts with the bases of the recognition site from the minor groove side are made by the residues located on the helix H4 (Supplementary Figure S1, Figure 2B and C). Two residues of each BsaWI subunit interact with the symmetry related CC:GG half-sites of the recognition sequence. The main chain O and the side chain Oδ1 atoms of N81 accept hydrogen bonds from the N2 atoms of guanines G9 and G8, respectively (Figure 2B). The side chain of K84 donates a hydrogen bond to the O2 atom of the complementary cytosine C7 from the central base pair. Additionally, K85 contacts the N3 atom of the outer adenine A5 of the same half-site (Figure 2C). Mutation K85A abolishes BsaWI-specific DNA binding and cleavage activities, indicating that K85 is important for the target recognition (Table 3).

**Table 3.** Characterization of the BsaWI mutants

Mutant	Function	Estimated M_w_, kDa	Oligomeric state^a^	*K*_d_, nM^b^	Ratio *k*_obs (1-site)/_*k*_obs (2-site)_^c^
wt		109.2	tetramer	2.9 ± 0.4	20.6
K85A	outer bp recognition	125.3	tetramer	228.8 ± 9.1	nh^d^
D145A	active site	92.3	tetramer	17.0 ± 4.7	nh^d^
D175A	active site	107.5	tetramer	6.1 ± 2.3	nh^d^
E128A	tetramerization	70.7	dimer	2.19 ± 0.4	17.9
R130A	tetramerization	76.7	dimer	3.9 ± 0.6	2.4
R259A	tetramerization	68.5	dimer	2.0 ± 0.3	14.4
		1396.6	oligomer		
W262A	tetramerization	321.0	decamer	2.7 ± 0.8	nh^d^
		196.7	hexamer		
		81.4	dimer		
E128A&R130A	tetramerization	69.9	dimer	2.1 ± 0.5	1.8
R259A&W262A	tetramerization	81.8	dimer	2.6 ± 0.6	2.2
R130A&R259A	tetramerization	77.5	dimer	2.7 ± 0.5	1.1
E128A&W262A	tetramerization	77.1	dimer	2.7 ± 0.7	2.2

^a^BsaWI gel filtration was performed using 5 μM concentration. Calculated theoretical M_w_ for BsaWI: monomer 31.9 kDa, dimer 63.8 kDa, tetramer 127.6 kDa. W262A eluted from the column at several peaks.

^b^*K*_d_ was determined using SP oligoduplex as described in ‘Materials and Methods’ section.

^c^*k*_obs_ ratio for 1-site and 2-site DNA substrates at optimal BsaWI (wt ant mutants) concentrations are presented.

^d^nh, no hydrolysis, since K85A, D145A, D175A and W262A mutants are inactive on λ DNA.

To recognize the CCGG tetranucleotide, the CCGG-family endonucleases use a conserved R-(D/E)R sequence motif (4,11). The corresponding residues of the BsaWI R-(D/E)R motif are R169, D170 and R171 (Figure 2D, Supplementary Figure S2A). In the structure of the pre-specific complex these residues are disordered, however the position of the modeled backbone atoms corresponds to the R-(D/E)R residues of NgoMIV in the superimposed structures suggesting that in the specific complex they would make similar contacts to the recognition sequence (Figure 2D, Supplementary Figure S2A). Loops between the strand S3 and the helix H7 and the N-termini of the helixes H7 and H8 are not well ordered and possess higher B-factors; these fragments might get more ordered upon the formation of the specific complex.

#### Active site

Restriction endonucleases of the CCGG-family possess a variation of the canonical PDX_10–30_(D/E)XK active site PDX_21–53_KX_12–13_E (4,11). The putative active site of BsaWI is composed of the residues E92, D145, K165 and D175. The first three residues overlay well with the corresponding active site residues of NgoMIV (Figure 2E, Supplementary Figure S2B). However, the second acidic residue of the PDXKXE motif of NgoMIV is E201, while BsaWI possess P178 at the corresponding position. Therefore we suggest that the second acidic residue of BsaWI is D175, which overlays with N197 of NgoMIV and is located close to the other predicted active site residues (Figure 2E, Supplementary Figure S2B). Despite the presence of Ca^2+^ ions in the crystallization buffer there is no electron density that could be interpreted as Ca^2+^ ion in the vicinity of the active site of BsaWI. Interestingly, the Nζ atom of the catalytic lysine K165 occupies the same position as Ca^2+^ ion in NgoMIV-DNA structure (PDB ID: 4ABT). Mutational analysis of the residues D145 and D175 confirmed their importance in the DNA cleavage (Table 3).

### BsaWI oligomeric state in solution

To determine the oligomeric state of BsaWI in solution we performed a gel filtration analysis. Gel filtration experiments performed at 5 μM loading protein concentration revealed that wt BsaWI elutes from the column as 112 kDa species, which is close to the calculated M_w_ of 128 kDa for a tetramer (Supplementary Figure S3A). The BsaWI–DNA complex with 30 bp SP oligoduplex (Supplementary Table S1) elutes from the column as 202 kDa species which is consistent with a BsaWI tetramer (experimental M_w_ = 112 kDa) bound to two DNA duplexes (expected mass 2 × 42 kDa, see Supplementary Figure S3A).

Gel filtration performed at different protein concentrations (0.25–25 μM) suggest that BsaWI exists in a dimer-tetramer-oligomer equilibrium (Supplementary Figure S3B, Supplementary Table S2). At low protein concentrations (0.25–1 μM) the dimeric form predominates, but dimers tend to form tetramers at higher concentrations (2.5–5 μM). At protein concentrations above10 μM BsaWI forms higher order aggregates (Supplementary Figure S3B).

To probe whether the contacts observed in the crystal (Figure 1C) are important for the BsaWI oligomerization in solution, we made a set of single and double mutants of the putative tetramerization/oligomerization interface residues E128, R130, R259 and W262 (Table 3). Gel filtration analysis of the tetramerization interface mutants performed at up to 25 μM protein concentration reveals that all mutants, except W262A, are dimers in solution (Supplementary Figure S3C and D, Table 3 and Supplementary Table S3). These data indicate that E128, R130 and R259 residues are important for the BsaWI tetramer/oligomer formation. Interestingly, the W262A mutation presumably disrupts the interface between two primary dimers and promotes formation of higher order BsaWI aggregates (Supplementary Figure S3D, Table 3).

### DNA binding assays

To explore the BsaWI interaction with DNA we performed EMSA experiments using oligoduplexes containing a 5′-ACCGGT-3′ recognition sequence (SP, see Supplementary Table S1) or lacking the target site (NSP). In the absence of divalent metal ions BsaWI does not bind to DNA (data not shown), however in the presence of Ca^2+^ ions BsaWI binds to specific DNA with K_d_ ∼3 nM (Supplementary Figure S4A). Interestingly, under these conditions BsaWI also binds to non-specific DNA with a relatively high affinity (K_d_ ∼10 nM), but the mobility of the non-specific complex is lower in comparison to the specific complex (Supplementary Figure S4B).

DNA binding experiments of the tetramerization mutants reveal that they bind to DNA with a similar affinity as wt BsaWI (Table 3). However, the mobility of the mutant complexes is higher compared to the wt enzyme tetramer bound to two DNA copies suggesting that BsaWI mutants are dimers bound to a single DNA molecule (Supplementary Figure S4C and D). Interestingly, the W262A mutant in a DNA bound-state forms multiple complexes with different mobility corresponding to distinct oligomeric aggregates consistent with gel filtration experiments (Supplementary Figure S3D and Table 3).

### BsaWI cleavage of 1- and 2-site DNA substrates

Gel filtration data suggest that the wt BsaWI tetramer binds two DNA duplexes in solution (Supplementary Figure S3A). REases which require binding of two cognate sites for an optimal catalytic activity cleave DNA substrates bearing two recognition sequences much faster than a single-site substrate (7,20,46). Therefore, we constructed DNA fragments containing one and two BsaWI target sites (termed 1-site substrate and 2-site substrate, respectively; Supplementary Table S1) and performed kinetic analysis of the BsaWI cleavage.

**Figure 3.**
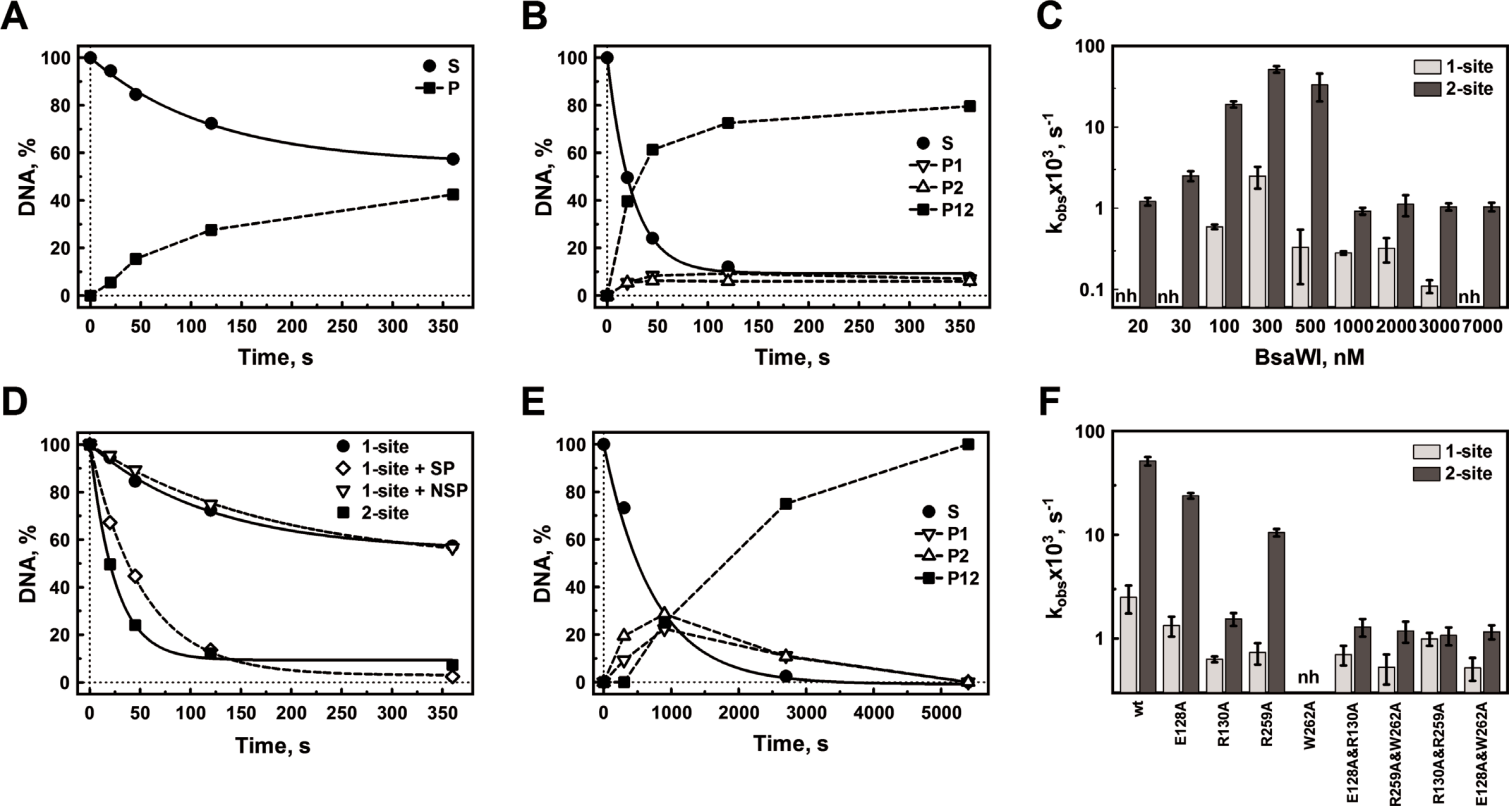
DNA cleavage by BsaWI. The BsaWI restriction enzyme on the1-site (**A**) and the 2-site (**B**) DNA substrates (Supplementary Table S1). (**C**) The values of the cleavage rate constants on the 1-site and the 2-site substrates at different wt BsaWI concentrations. (A–C) The reactions at 37°C contained the 1-site or the 2-site substrates (at 10 nM) and 300 nM (A and B) and 20–7000 nM (C) wt BsaWI in the Reaction buffer (see ‘Materials and Methods’ section). (**D**) The cleavage of the 1-site substrate (10 nM) in the presence of 300 nM of the cognate (SP) or the non-cognate (NSP) oligoduplex by 300 nM BsaWI. (**E**) The BsaWI double mutant E128&R130A (1000 nM) on the 2-site DNA substrate (10 nM). (**F**) The cleavage rate constants of the tetramerization mutants on the 1-site and the 2-site substrates, determined at the optimal enzyme concentrations (see ‘Materials and Methods’ section). S—substrate, P—product (the 1-site substrate cleaved at the single site), P1 and P2—cleaved at the first (S1) and the second site (S2) of the 2-site substrate, respectively, P12—the 2-site substrate cleaved at both sites, nh—no hydrolysis (wt BsaWI under the experimental conditions does not cleave DNA, the mutant W262A is inactive).

The BsaWI reaction rates on the 1-site and the 2-site DNA substrates were measured within 20–7000 nM range of the enzyme concentrations (Figure 3A–C). The experimentally established optimal enzyme concentrations are 100–300 nM for the 1-site and 100–500 nM for the 2-site substrate, respectively (Figure 3A and B). Interestingly, the enzyme is active in a narrow range of concentrations: at low (<100 nM) and at very high (>500 nM) concentrations the reaction rate drops dramatically. The cleavage rate ratio for the 2-site and the 1-site substrates is ∼20 (at optimal 300 nM protein concentration), indicating that BsaWI prefers the 2-site substrate. Moreover, cleavage of the 1-site substrate is stimulated *in trans* by addition of the specific oligoduplex containing the recognition sequence (Figure 3D). The cleavage rate of the 1-site substrate in the presence of the specific oligoduplex is similar to that of the 2-site substrate. Therefore, the tetrameric BsaWI shows its optimal catalytic activity after binding two copies of the target sequence either *in cis* or *in trans*. In the case of the 2-site substrate DNA cleavage occurs at both sites in a concerted manner (Figure 3B). To estimate whether both strands of the recognition site are cleaved simultaneously we performed cleavage experiments of a hairpin HP substrate (Supplementary Table S1, Supplementary Figure S5). The cleavage data show that both DNA strands of the single recognition site are also cleaved concertedly.

Next, we explored the ability of the dimeric mutants to cleave the 1-site and the 2-site DNA (Supplementary Table S4, Figure 3E and F). The cleavage experiments under single turnover conditions (10 nM DNA and 100–700 nM protein) show that the single mutants E128A and R259A still prefer the 2-site versus the 1-site substrate (the cleavage rate ratio of the 2-site and the 1-site substrates is ∼18- and ∼15-fold, respectively) (Table 3). These results suggest that the E128A and R259A mutants, despite of a compromised salt bridge (Figure 1C), still are able to form tetramers under the cleavage conditions. In contrast, the R130A mutant cleaves the 1-site and the 2-site substrates slowly, indicating that it functions as a dimer. In the case of the double mutants E128A&R130A, R259A&W262A, R130A&R259A and E128A&W262A, DNA cleavage is observed at much higher protein concentrations, (1–3 μM), and the cleavage rate ratio for the 2-site/1-site substrates is only approximately two-fold (Table 3) presumably due to a statistical factor. In contrast to the wt BsaWI that shows a concerted cleavage at both sites (Figure 3B), the E128A&R130A mutant cleaves both targets of the 2-site substrate independently (Figure 3E). The tetramerization interface mutants R130A, E128A&R130A, R259A&W262A, R130A&R259A, E128A&W262A behave as dimers on both 1-site and 2-site substrates, however their cleavage activity is very low. Taken together these data suggest that cleavage of the 1-site DNA by wt BsaWI occurs in the synaptic complex, where the BsaWI tetramer is bound to two DNA targets *in trans*.

## DISCUSSION

### Pre-specific complex

We solved the crystal structure of BsaWI bound to the specific DNA (Figure 1A). BsaWI in the crystal is captured in an ‘open’ configuration (the pre-specific complex) in which only minor groove contacts to the target site are established (Figures 1A, 2B and C). The R-(D/E)R motif residues, which are supposed to recognize CCGG from the major groove side, are poorly ordered and located far away from the DNA bases (Figure 2D). The crystal contacts result in an indefinite protein chain and presumably interfere with a formation of the proper catalytically competent tetramer (Figure 1B).

The dimer structure in the BsaWI pre-specific complex is similar to the C-terminal domain dimer of EcoRII (EcoRII-C) in the DNA-free form. Apo-EcoRII-C dimer is in an ‘open’ configuration in which the dimer interface is formed only by the contacts of the N-terminal helices while the C-terminal catalytic cores do not contact each other (Supplementary Figure S6) (13). In the DNA-bound form of EcoRII-C dimer and structurally similar complexes of Ecl18kI and PspGI, protein adopts a ‘closed’ configuration and completely encircles DNA (5,9,12). In the ‘closed’ form both the N-terminal and C-terminal domain contacts contribute to the dimer interface. In the case of Ecl18kI and EcoRII-C, the interface at the N-terminal region is about two- to three-fold larger compared to the interface at the catalytic core (1340 versus 440Å^2^ in Ecl18kI, 800 versus 420 Å^2^ in EcoRII-C) while in PspGI the N-terminal domain interface makes smaller contribution than the C-terminal catalytic domains (410 versus 1230 Å^2^). Restriction endonuclease BsoBI specific for the 5′-CYCGRG-3′ sequence also shows a bipartite dimer interface and completely enwraps DNA (47). To allow DNA entry either the N- or C-terminal domain the dimer interface has to open. Since the N-terminal domain interface is significantly larger than the interface at the catalytic core (1750 versus 500 Å^2^), authors speculate that C-terminal catalytic domains move apart opening the gate for DNA binding (47).

We hypothesize that in the crystal BsaWI primary dimer is an ‘open’ configuration because the crystal contacts at the C-domain interfere with the C-terminal domain closure and transition to the ‘closed’ form observed in EcoRII-C, Ecl18kI, PspGI and BsoBI DNA complexes. In the ‘closed’ form BsaWI would engage into the base specific major groove interactions with the CCGG tetranucleotide via R_169_D_170_R_171_ residues located on the structurally conserved recognition helix. We also propose that transition between ‘closed’ and ‘open’ configurations should also occur in Ecl18kI and PspGI. In Ecl18kI opening of a dimer probably occurs at the C-terminal catalytic domain interface, similar to EcoRII-C and BsaWI. PspGI presumably opens at the N-terminal interface to accommodate DNA, since the interface at the catalytic C-terminal domain is much larger.

### Recognition of the outer A:T/T:A base pairs

In the pre-specific complex BsaWI makes no contacts to the outer base pair in the major groove, therefore we can only speculate about the recognition of the outer base pair by BsaWI. From the minor groove side K85 donates hydrogen bond to the N3 atom of the outer adenine base (Figure 2C). To recognize an alternative thymine base, K85 could make hydrogen bond to the O2 atom (Supplementary Figure S7A). The similar hydrogen bond could be made with the O2 atom of the cytosine base instead of thymine; however, in that case the side chain of K85 would clash with the N2 atom of the complementary guanine base (Supplementary Figure S7B). A guanine base in place of the adenine would also clash with K85 (Supplementary Figure S7C). Therefore the recognition of the outer base pair may depend solely on the K85 contacts. The mutation K85A abolishes DNA binding and cleavage activities of BsaWI (Table 3). One can not exclude that the recognition of the outer base pair also involves contacts from the major groove; however, the available data can not pinpoint the residues that could participate in this recognition. To elucidate this, the structure of the specific BsaWI–DNA complex is still required.

Other CCGG-family enzymes use the residues from the analogous structural elements for the recognition of the bases in the minor groove outside the CCGG tetranucleotide in the 6 and 8 bp recognition sites. In the NgoMIV (recognition sequence 5′-GCCGGC-3′) the Q63 residue makes contact with the cytosine base of the outer G:C base pair; in Bse634I (5′-RCCGGY-3′) residue N73 makes contacts with O_2_ atoms of pyrimidines; in SgrAI (5′-CRCCGGYG-3′) K96 makes hydrogen bond to N3 atom of G from the outer C:G base pair in the minor groove (7,8,11). Differently from NgoMIV and Bse634I that recognize 6 bp sequences, SgrAI interacts with 8 bp DNA target; therefore the outer base pair of its recognition sequence is further away from the CCGG tetranucleotide in respect to the NgoMIV and Bse634I sequences. Minor groove contacts to the outer base pair of the 6 bp target are conserved (Supplementary Figure S7D) in BsaWI (K85), NgoMIV (Q63) and Bse634I (N73) but in SgrAI a structurally equivalent K96 residue does not make direct contacts to the R:Y base pair which coincides with the outer base pair in the BsaWI/NgoMIV/Bse634I recognition sequences. It cannot be excluded that conserved K96 residue contributes to the indirect readout of the R:Y base pair by unstacking the G base from the preceding Y base (8). Another conserved position for the base-specific contacts in the major groove of Bse634I/NgoMIV/SgrAI is located within the R-(D/E)R motif that recognizes the CCGG tetranucleotide. P203/S192/S247 of Bse634I/NgoMIV/SgrAI, respectively, are situated between the first arginine and the following aspartate residue in the motif (Supplementary Figure S7E) (7,8,11). In contrast, the R-(D/E)R motif in BsaWI is continuous (R_169_D_170_R_171_) and lacks such residue suggesting that BsaWI may use different structural elements for the recognition of the outer base pair. NgoMIV, which recognizes non-degenerated sequence, also possesses additional determinants for the outer base pair recognition (residues D34 and R227) (7).

### BsaWI is optimally active as a tetramer bound to two DNA targets

Gel filtration experiments and the crystal contacts show that BsaWI can exist as a dimer, a tetramer or a higher order oligomer (Supplementary Figure S3B). In the presence of specific DNA BsaWI forms a tetramer that binds two DNA molecules (Supplementary Figure S3A). The synaptic complex, where the BsaWI tetramer is bound to two recognition sequences, is optimal for the catalytic activity (Figure 3A–C). In the synaptic complex cleavage of the 2-site DNA is fast and occurs concertedly at both sites resulting in the simultaneous cleavage of four phosphodiester bonds (Figure 3B, Supplementary Figure S5). Mutations R130A, E128A&R130A, R259A&W262A, R130A&R259A and E128A&W262A at the tetramerization interface observed in the crystal result in the dimeric variants that bind a single DNA molecule and exhibit slow and non-concerted cleavage of the 2-site substrate (Table 3). The W262A mutation at the tetramerization interface promotes protein oligomerization even at low protein concentrations and yields a catalytically inactive variant (Table 3, Figure 3F). Wt BsaWI is also prone to oligomerization and shows no DNA cleavage activity at high protein concentrations, (Supplementary Table S2) (Figure 3C) suggesting that oligomeric aggregates are unable to cleave DNA.

**Figure 4.**
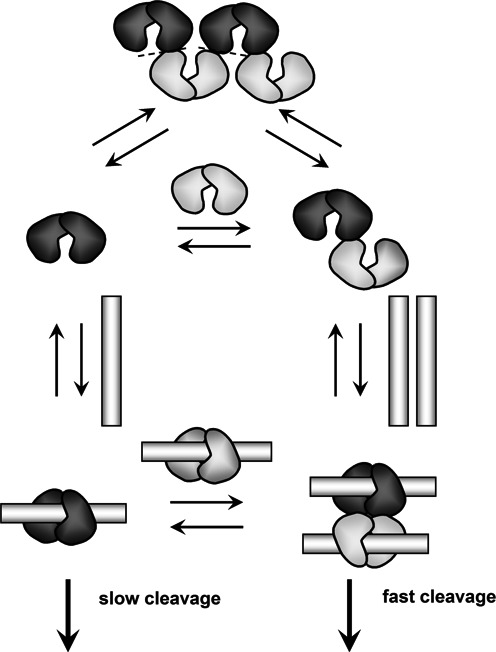
Mechanism of action of BsaWI. The cartoons depict the schematic mechanism of the BsaWI DNA cleavage. The primary dimers are colored light gray and dark gray, respectively. Interfaces of the possible oligomer dissociation are marked by dotted lines. BsaWI exists in solution in dimer-tetramer-oligomer equilibrium. Both dimer and ‘open’ configuration tetramer can bind to DNA. The optimal DNA cleavage complex is a tetramer bound to two DNA targets; it cleaves both DNA targets at both strands fast. The dimer is also able to cleave DNA, but at much slower rate. At high concentrations BsaWI oligomers are able to bind DNA but do not cleave it.

Taking into consideration structural and biochemical data, we postulate the following BsaWI mechanism (Figure 4). The wt protein in solution exists in a dimer-tetramer-oligomer equilibrium depending on the protein concentration (Supplementary Figure S3B). The crystal contacts observed in the BsaWI oligomers presumably contribute to protein oligomerization in solution. BsaWI higher order aggregates exist in equilibrium with dimers or tetramers. Disruption of the tetramerization interface in R130A, E128A&R130A, R259A&W262A, R130A&R259A and E128A&W262A mutants yields dimeric variants that bind a single DNA target and cut it slowly. In the case of wt BsaWI, two DNA-bound dimers may assemble into a catalytically competent tetrameric synaptic complex that cleaves fast and concertedly both DNA strands at both sites.

Type II restriction endonucleases, which cleave palindromic DNA sequences, act as monomers, dimers, tetramers or higher order oligomers (Supplementary Table S5). Interestingly, distinct oligomeric forms are characteristic for the CCGG-family enzymes (Supplementary Table S5). PspGI as other orthodox Type IIP enzymes is functional as a dimer and cleaves phosphodiester bonds in both DNA strands at single target site. Type IIF enzymes, exemplified by Cfr10I, Bse634I, NgoMIV, are homotetramers both in apo- and DNA-bound forms; they bind and cleave two target sites in a concerted manner (7,10,14–16,18,20,46). Ecl18kI and SgrAI exist in dimer-tetramer or dimer-oligomer equilibrium and represent intermediate variants between dimeric and tetrameric enzymes. Interestingly, their DNA cleavage mechanisms differ. Ecl18kI is a dimer in solution but forms catalytically competent tetramers upon DNA binding (4,20). SgrAI is also a dimer in the apo-form, however after binding to a specific target site it forms so called ‘run-on’ oligomers, which show relaxed sequence specificity (8,15,48,49). The suggested mechanism for BsaWI shares common features both with dimeric Ecl18kI/SgrAI and tetrameric Type IIF enzymes NgoMIV/Bse634I/Cfr10I and SfiI. In solution apo-BsaWI exists either as a dimer similar to Ecl18kI/SgrAI proteins, or a tetramer, resembling NgoMIV/Bse634I/Cfr10I enzymes. The BsaWI tetramer like other Type IIF restriction endonucleases requires binding of two target sites for the optimal catalytic activity. BsaWI, like SgrAI, also forms oligomers at high protein concentrations, however, differently from SgrAI, BsaWI oligomers are catalytically inactive. Structural and biochemical data presented here provide new insights into the structure-function relationships in the family of restriction enzymes.

## ACCESSION NUMBER

Coordinates and structure factors of BsaWI–DNA complex are deposited under PDB ID: 4ZSF.

## Supplementary Material

SUPPLEMENTARY DATA

## References

[1] Roberts R.J., Belfort M., Bestor T., Bhagwat A.S., Bickle T.A., Bitinaite J., Blumenthal R.M., Degtyarev S., Dryden D.T., Dybvig K. (2003). A nomenclature for restriction enzymes, DNA methyltransferases, homing endonucleases and their genes. Nucleic Acids Res..

[2] Pingoud A., Fuxreiter M., Pingoud V., Wende W. (2005). Type II restriction endonucleases: structure and mechanism. Cell Mol. Life Sci..

[3] Pingoud V., Kubareva E., Stengel G., Friedhoff P., Bujnicki J.M., Urbanke C., Sudina A., Pingoud A. (2002). Evolutionary relationship between different subgroups of restriction endonucleases. J. Biol. Chem..

[4] Tamulaitis G., Solonin A.S., Siksnys V. (2002). Alternative arrangements of catalytic residues at the active sites of restriction enzymes. FEBS Lett..

[5] Bochtler M., Szczepanowski R.H., Tamulaitis G., Grazulis S., Czapinska H., Manakova E., Siksnys V. (2006). Nucleotide flips determine the specificity of the Ecl18kI restriction endonuclease. EMBO J..

[6] Bozic D., Grazulis S., Siksnys V., Huber R. (1996). Crystal structure of Citrobacter freundii restriction endonuclease Cfr10I at 2.15 A resolution. J. Mol. Biol..

[7] Deibert M., Grazulis S., Sasnauskas G., Siksnys V., Huber R. (2000). Structure of the tetrameric restriction endonuclease NgoMIV in complex with cleaved DNA. Nat. Struct. Biol..

[8] Dunten P.W., Little E.J., Gregory M.T., Manohar V.M., Dalton M., Hough D., Bitinaite J., Horton N.C. (2008). The structure of SgrAI bound to DNA; recognition of an 8 base pair target. Nucleic Acids Res..

[9] Golovenko D., Manakova E., Tamulaitiene G., Grazulis S., Siksnys V. (2009). Structural mechanisms for the 5′-CCWGG sequence recognition by the N- and C-terminal domains of EcoRII. Nucleic Acids Res..

[10] Grazulis S., Deibert M., Rimseliene R., Skirgaila R., Sasnauskas G., Lagunavicius A., Repin V., Urbanke C., Huber R., Siksnys V. (2002). Crystal structure of the Bse634I restriction endonuclease: comparison of two enzymes recognizing the same DNA sequence. Nucleic Acids Res..

[11] Manakova E., Grazulis S., Zaremba M., Tamulaitiene G., Golovenko D., Siksnys V. (2012). Structural mechanisms of the degenerate sequence recognition by Bse634I restriction endonuclease. Nucleic Acids Res..

[12] Szczepanowski R.H., Carpenter M.A., Czapinska H., Zaremba M., Tamulaitis G., Siksnys V., Bhagwat A.S., Bochtler M. (2008). Central base pair flipping and discrimination by PspGI. Nucleic Acids Res..

[13] Zhou X.E., Wang Y., Reuter M., Mucke M., Kruger D.H., Meehan E.J., Chen L. (2004). Crystal structure of type IIE restriction endonuclease EcoRII reveals an autoinhibition mechanism by a novel effector-binding fold. J. Mol. Biol..

[14] Siksnys V., Skirgaila R., Sasnauskas G., Urbanke C., Cherny D., Grazulis S., Huber R. (1999). The Cfr10I restriction enzyme is functional as a tetramer. J. Mol. Biol..

[15] Bilcock D.T., Daniels L.E., Bath A.J., Halford S.E. (1999). Reactions of type II restriction endonucleases with 8-base pair recognition sites. J. Biol. Chem..

[16] Embleton M.L., Siksnys V., Halford S.E. (2001). DNA cleavage reactions by type II restriction enzymes that require two copies of their recognition sites. J. Mol. Biol..

[17] Pingoud V., Conzelmann C., Kinzebach S., Sudina A., Metelev V., Kubareva E., Bujnicki J.M., Lurz R., Luder G., Xu S.Y. (2003). PspGI, a type II restriction endonuclease from the extreme thermophile Pyrococcus sp.: structural and functional studies to investigate an evolutionary relationship with several mesophilic restriction enzymes. J. Mol. Biol..

[18] Zaremba M., Sasnauskas G., Urbanke C., Siksnys V. (2005). Conversion of the tetrameric restriction endonuclease Bse634I into a dimer: oligomeric structure-stability-function correlations. J. Mol. Biol..

[19] Tamulaitis G., Sasnauskas G., Mucke M., Siksnys V. (2006). Simultaneous binding of three recognition sites is necessary for a concerted plasmid DNA cleavage by EcoRII restriction endonuclease. J. Mol. Biol..

[20] Zaremba M., Owsicka A., Tamulaitis G., Sasnauskas G., Shlyakhtenko L.S., Lushnikov A.Y., Lyubchenko Y.L., Laurens N., van den Broek B., Wuite G.J. (2010). DNA synapsis through transient tetramerization triggers cleavage by Ecl18kI restriction enzyme. Nucleic Acids Res..

[21] Tamulaitis G., Zaremba M., Szczepanowski R.H., Bochtler M., Siksnys V. (2007). Nucleotide flipping by restriction enzymes analyzed by 2-aminopurine steady-state fluorescence. Nucleic Acids Res..

[22] Zheng L., Baumann U., Reymond J.L. (2004). An efficient one-step site-directed and site-saturation mutagenesis protocol. Nucleic Acids Res..

[23] Van Duyne G.D., Standaert R.F., Karplus P.A., Schreiber S.L., Clardy J. (1993). Atomic structures of the human immunophilin FKBP-12 complexes with FK506 and rapamycin. J. Mol. Biol..

[24] Leslie A.G.W. (2006). The integration of macromolecular diffraction data. Acta Crystallogr. D Biol. Crystallogr..

[25] CCP4 (1994). The CCP4 suite: programs for protein crystallography. Acta Crystallogr. D Biol. Crystallogr..

[26] Panjikar S., Parthasarathy V., Lamzin V.S., Weiss M.S., Tucker P.A. (2005). Auto-rickshaw: an automated crystal structure determination platform as an efficient tool for the validation of an X-ray diffraction experiment. Acta Crystallogr. D Biol. Crystallogr..

[27] Sheldrick G.M., Hauptman H. A., Weeks C. M., Miller R., Uson I., MGRaE Arnold (2001). Ab initio phasing. International Tables for Macromolecular Crystallography.

[28] Schneider T.R., Sheldrick G.M. (2002). Substructure solution with SHELXD. Acta Crystallogr. D Biol. Crystallogr..

[29] Hao Q. (2004). ABS: a program to determine absolute configuration and evaluate anomalous scatterer substructure. J. Appl. Cryst..

[30] Sheldrick G.M. (2002). Macromolecular phasing with SHELXE. Z. Kristallogr..

[31] Pannu N.S., McCoy A.J., Read R.J. (2003). Application of the complex multivariate normal distribution to crystallographic methods with insights into multiple isomorphous replacement phasing. Acta Crystallogr. D Biol. Crystallogr..

[32] Terwilliger T.C. (2000). Maximum-likelihood density modification. Acta Crystallogr. D Biol. Crystallogr..

[33] Morris R.J., Zwart P.H., Cohen S., Fernandez F.J., Kakaris M., Kirillova O., Vonrhein C., Perrakis A., Lamzin V.S. (2004). Breaking good resolutions with ARP/wARP. J. Synchrotron Rad..

[34] Vagin A., Teplyakov A. (2010). Molecular replacement with MOLREP. Acta Crystallogr. D Biol. Crystallogr..

[35] Murshudov G., Vagin A., Dodson E. (1996). Application of maximum likelihood refinement in the refinement of protein structures. Proceedings of Daresbury Study Weekend.

[36] Emsley P., Cowtan K. (2004). Coot: model-building tools for molecular graphics. Acta Crystallogr. D Biol. Crystallogr..

[37] Afonine P.V., Grosse-Kunstleve R.W., Echols N., Headd J.J., Moriarty N.W., Mustyakimov M., Terwilliger T.C., Urzhumtsev A., Zwart P.H., Adams P.D. (2012). Towards automated crystallographic structure refinement with phenix.refine. Acta Crystallogr. D Biol. Crystallogr..

[38] Painter J., Merritt E.A. (2006). TLSMD web server for the generation of multi-group TLS models. J. Appl. Cryst..

[39] Krissinel E., Henrick K. (2007). Inference of macromolecular assemblies from crystalline state. J. Mol. Biol..

[40] Luscombe N.M., Laskowski R.A., Thornton J.M. (1997). NUCPLOT: a program to generate schematic diagrams of protein-nucleic acid interactions. Nucleic Acids Res..

[41] Kraulis P. (1991). Molscript-a program to produce both detailed and schematic plots of protein structures. J. Appl. Cryst..

[42] Merritt E.A., Murphy M.E. (1994). Raster3D Version 2.0 A program for photorealistic molecular graphics. Acta Crystallogr. D Biol. Crystallogr..

[43] Tamulaitis G., Mucke M., Siksnys V. (2006). Biochemical and mutational analysis of EcoRII functional domains reveals evolutionary links between restriction enzymes. FEBS Lett..

[44] Yoshioka K. (2002). KyPlot—a user-oriented tool for statistical data analysis and visualization. Comp. Stat..

[45] Holm L., Rosenstrom P. (2010). Dali server: conservation mapping in 3D. Nucleic Acids Res..

[46] Wentzell L.M., Nobbs T.J., Halford S.E. (1995). The SfiI restriction endonuclease makes a four-strand DNA break at two copies of its recognition sequence. J. Mol. Biol..

[47] van der Woerd M.J., Pelletier J.J., Xu S., Friedman A.M. (2001). Restriction enzyme BsoBI-DNA complex: a tunnel for recognition of degenerate DNA sequences and potential histidine catalysis. Structure.

[48] Park C.K., Stiteler A.P., Shah S., Ghare M.I., Bitinaite J., Horton N.C. (2010). Activation of DNA cleavage by oligomerization of DNA-bound SgrAI. Biochemistry.

[49] Lyumkis D., Talley H., Stewart A., Shah S., Park C.K., Tama F., Potter C.S., Carragher B., Horton N.C. (2013). Allosteric regulation of DNA cleavage and sequence-specificity through run-on oligomerization. Structure.

