# Supplementary material for: Functional significance of protein assemblies predicted by the crystal structure of the restriction endonuclease BsaWI
Source: Nucleic Acids Res. 2015 Aug 3;43(16):8100–10. doi: 10.1093/nar/gkv768 (PMC4652773; doi:10.1093/nar/gkv768)
Supplement: SUPPLEMENTARY DATA [file supp_43_16_8100__index.html]

Functional significance of protein assemblies predicted by the crystal structure of the restriction endonuclease BsaWI — Functional significance of protein assemblies predicted by the crystal structure of the restriction endonuclease BsaWI — SUPPLEMENTARY DATA 

# Functional significance of protein assemblies predicted by the crystal structure of the restriction endonuclease BsaWI

## SUPPLEMENTARY DATA

- SUPPLEMENTARY DATA
