# Supplementary material for: Functional significance of protein assemblies predicted by the crystal structure of the restriction endonuclease BsaWI
Source: Nucleic Acids Res. 2015 Aug 3;43(16):8100–10. doi: 10.1093/nar/gkv768 (PMC4652773; doi:10.1093/nar/gkv768)
Supplement: SUPPLEMENTARY DATA [file supp_gkv768_nar-01625-h-2015-File006.pdf]

Supplementary data to:

Functional significance of protein assemblies predicted by the crystal structure of the restriction endonuclease BsaWI

Gintautas Tamulaitis[1,†], Marius Rutkauskas[1,2,†], Mindaugas Zaremba[1], Saulius Grazulis[1], Giedre Tamulaitiene[1,*] and Virginijus Siksnys[1,*]
[1] Department of Protein–DNA Interactions, Institute of Biotechnology, Vilnius University, Graiciuno 8, LT-02241, Vilnius, Lithuania
[2] present address: Institute for Molecular Cell Biology, Westfälische Wilhelms-Universität Münster, 48149 Münster, Germany.

[†] The authors wish it to be known that, in their opinion, the first two authors should be regarded as joint First Authors.
* To whom correspondence should be addressed. Tel: +370-5-2602111; Fax: +370-5-2602116; Email: eigie@ibt.lt
Correspondence may also be addressed to Virginijus Siksnys. Tel. +370-5-2602108; Fax: +370-5-2602116; Email: siksnys@ibt.lt

Supplementary Table S1. DNA substrates used in this study

Substrate	Used for	Sequence/scheme
SP14<br>(specific)	crystallization	5′-CTCG**ACCGGT**CGAG-3′<br>3′-GAGC**TGGCCA**GCTC-5′
SP<br>(specific)	DNA binding,<br>stimulation of cleavage	5′-GCCTTTCGGTTT**ACCGGT**GTCATTCCGCTG-3′<br>3′-CGGAAAGCCAAA**TGGCCA**CAGTAAGGCGAC-5′
NSP<br>(nonspecific)	DNA binding,<br>stimulation of cleavage	5′-AATGGGCTCGCACGCCTGGTATTATCGATTGTA-3′<br>3′-TTACCCGAGCGTGCGGACCATAATAGCTAACAT-5′
HP<br>(specific)	cleavage	5′-AGACCCACGCTC**ACCGGT**GAGAGATTACGT\<br>3′-TCTGGGTGCGAG**TGGCCA**CTCTCTAATGAT/
1-site	cleavage	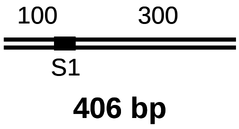
2-site	cleavage	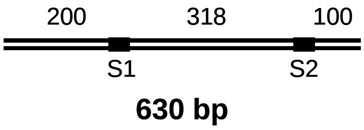

BsaWI recognition sequence is underlined or depicted as black block. S1 and S2 stand for site 1 and site 2 (both are 5’-ACCGGT-3’), respectively.

Supplementary Table S2. Wt BsaWI gel filtration at different protein concentrations.

BsaWI concentration, μM	Estimated M_w, kDa	Oligomeric state[a]
0.25	76.7	dimer
0.50	78.7	dimer
1.0	81.0	dimer
2.5	88.0	dimer-tetramer
5	109.2	tetramer
10	150.2	tetramer-hexamer
25	200.6	hexamer

[a] Calculated theoretical M_w for BsaWI – monomer 31.9 kDa, dimer 63.8 kDa, tetramer 127.6 kDa, hexamer 191.4 kDa.

Supplementary Table S3. Mutant BsaWI gel filtration at different protein concentrations.

Mutant	BsaWI concentration, μM	Estimated M_w, kDa	Oligomeric state[a]
E128A&R130A	5	65.9	dimer
	25	64.0	dimer
R259A&W262A	5	70.1	dimer
	25	70.7	dimer
R130A&R259A	5	70.8	dimer
	25	66.6	dimer
E128A&W262A	5	72.2	dimer
	25	67.6	dimer

[a] Calculated theoretical M_w for BsaWI dimer is 63.8 kDa.

Supplementary Table S4. Cleavage rate constants for wt and mutant BsaWI.

Mutant	Optimal concentration, nM	$k_{obs\ (1\text{-}site)}$, x10^3 s^{-1}	$k_{obs\ (2\text{-}site)}$, x10^3 s^{-1}
wt	300	2.5±0.8	51.4±4.8
E128A	1000	1.3±0.3	23.8±1.4
R130A	1000	0.6±0.1	1.5±0.2
R259A	3000	0.7±0.2	10.5±0.9
W262A	-	nh[a]	nh[a]
E128A&R130A	300-7000	0.7±0.1	1.3±0.2
R259A&W262A	300-7000	0.5±0.2	1.2±0.3
R130A&R259A	300-7000	1.0±0.1	1.1±0.2
E128A&W262A	300-3000	0.5±0.1	1.2±0.2

[a] nh – no hydrolysis

Supplementary Table S5. Variety of the oligomeric forms of the Type II PD-(D/E)XK restriction endonucleases recognizing symmetric sequences

Oligomeric form[a]	DNA cleavage mechanism	Examples
**Monomer**	• one active site cleaves both strands sequentially	BcnI (50,51)
**Dimer-1 site**	• dimer binds to a single DNA target<br>• two active sites cleave both strands of a single target site	EcoRII-C (19)<br>PspGI (17)
**Dimer-2 sites**	• back-to-back dimer composed of two monomers bound to a single site binds to two DNA targets, both strands of the targets are cleaved sequentially	HinP1I (52,53)
**Dimer-tetramer**	• dimer in the apo form, in the presence of DNA forms tetramers bound to two sites<br>• optimal activity requires binding to two target sites<br>• cleave both targets with a different rate	Ecl18kI (4,5,20) (M.Z., unpublished data)
**Dimer-tetramer-oligomer**	• dimer-tetramer-oligomer in the apo form, in the presence of DNA forms tetramers bound to two sites<br>• optimal activity requires binding to two target sites<br>• cleave both strands of both targets in a concerted manner	BsaWI (this study)
**Dimer-oligomer**	• dimer in the apo form and in the presence of DNA, in the presence of cognate DNA forms oligomers which may bind to the secondary sites<br>• optimal activity requires binding to two target sites; cleave both targets in a concerted manner<br>• cleaves DNA at secondary sites if a cognate site is present ("run-on" oligomer)	SgrAI (8,15,48,49,54)
**Tetramer**	• tetramer in the apo form and in the presence of DNA<br>• optimal activity requires binding to two target sites<br>• cleave both targets in a concerted manner	Cfr10I (14,16,55)<br>Bse634I (10,18)<br>NgoMIV (7)<br>SfiI (46)

[a] oligomeric forms found in CCGG-family restriction endonucleases are shown on grey background

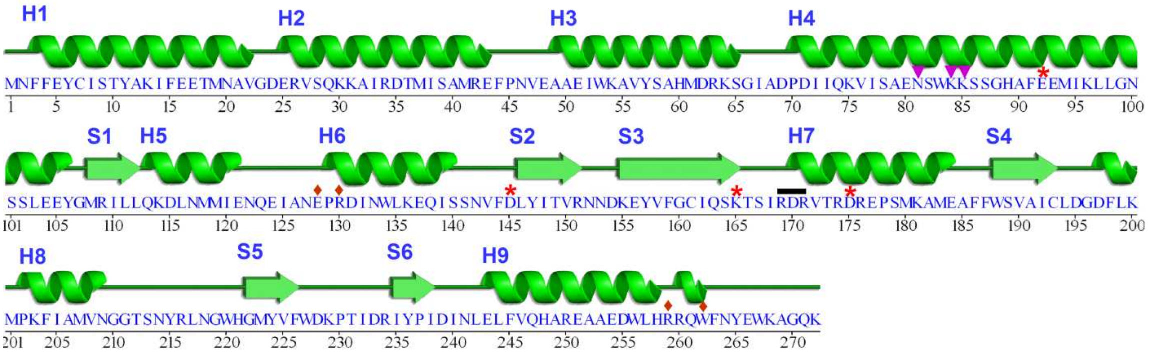

Figure S1. Topology diagram of BsaWI. Active site residues are marked by red asterisks. R-(D/E)R motif is shown by black line. Magenta triangles denote residues involved in contacts with DNA bases from the minor groove. Residues that are involved in tetramerization contacts are marked by orange diamonds. The diagram was prepared using PDBsum (http://www.ebi.ac.uk/pdbsum/).

A

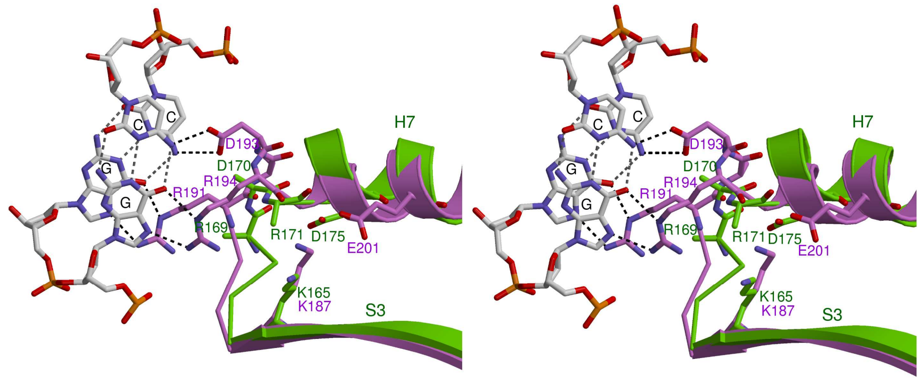

B

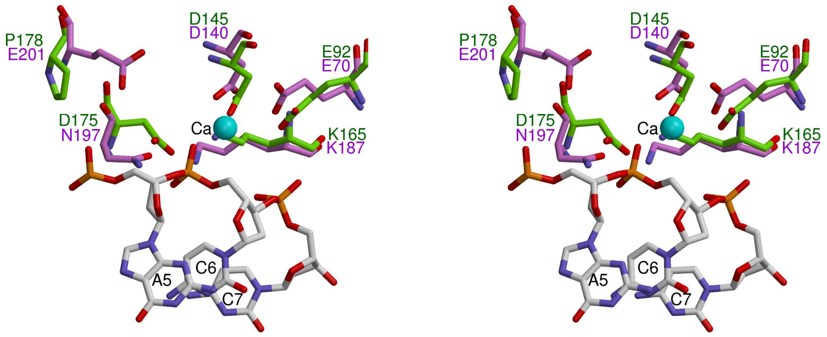

Figure S2. Stereo views of BsaWI CCGG recognition motif and active site. (A) The NgoMIV R-(D/E)R residues (violet, 4ABT) overlaid with the BsaWI putative CCGG recognition residues (green). Structural elements of NgoAVII and BsaWI (S3 and H7) bearing the catalytic lysine and glutamate/aspartate residues are also shown. DNA is from the NgoMIV complex. (B) The active site residues of BsaWI (green) overlaid with the active site of NgoMIV (violet, PDB ID 4ABT). DNA and Ca^{2+} ion (cyan sphere) from the NgoMIV-DNA structure are shown.

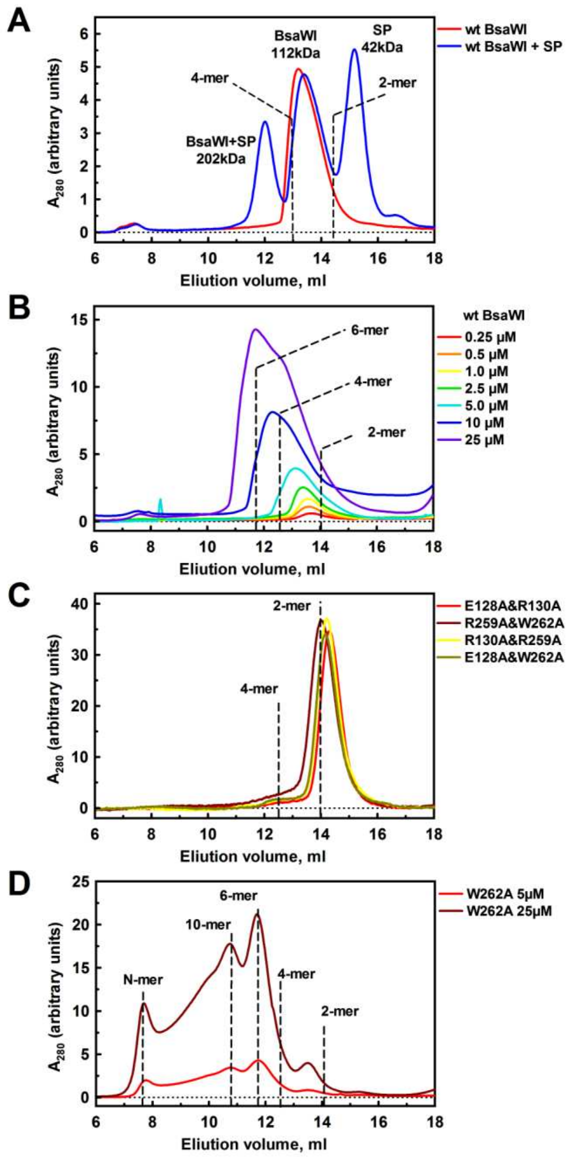

Figure S3. Gel filtration of BsaWI. (A) Elution profiles of wt BsaWI (5 μM loading concentration) and the BsaWI-DNA (5 μM protein and 1 μM SP DNA) complex. Experimentally determined Mw's are shown on the graph. Gel filtration was carried out as described in 'Materials and Methods'. Apo BsaWI elutes from the column as a 112 kDa protein whith a molecular weight close to the calculated M_w of the tetramer (calculated MW 4x31.9 kDa=127.6 kDa). SP DNA elutes from the column at a volume corresponding to 42 kDa possibly due to the elongated form of the molecule (calculated MW is 19 kDa). BsaWI-DNA complex elutes from the column as a 202 kDa species suggesting that one BsaWI tetramer binds two DNA duplexes (112 kDa+2x42 kDa=196 kDa). (B) Elution profiles of wt BsaWI at 0.25-25 μM loading concentrations. (C) Elution profiles of the tetramerization mutants at 25 μM concentration. D) Elution profiles of the W262A BsaWI at 5 and 25 μM concentrations. (A-D) Dotted lines represent the calculated elution positions of the BsaWI dimer, tetramer and/or higher oligomeric forms.

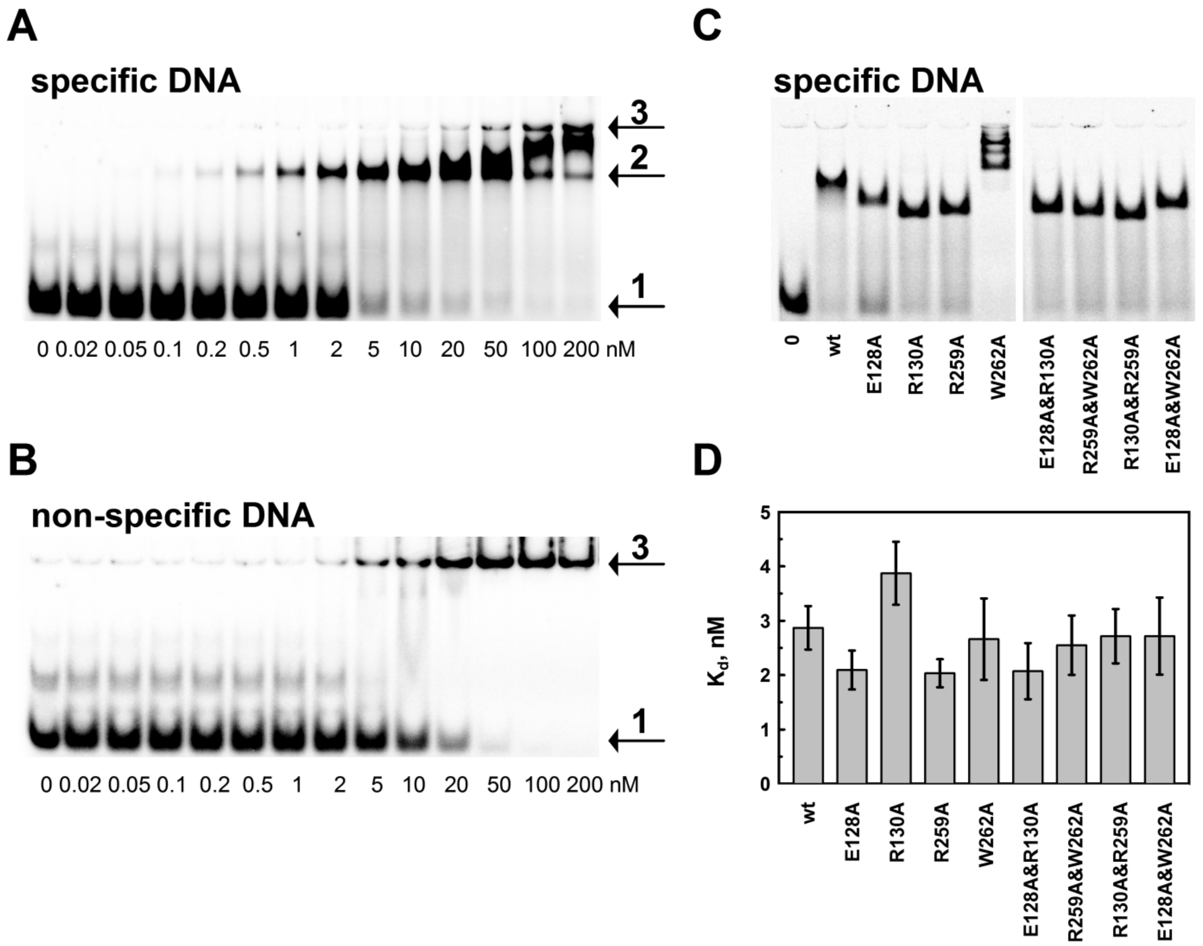

Figure S4. DNA binding by BsaWI. (A-B) Gel shift assay of wt BsaWI binding to cognate SP DNA oligoduplex (A) and non-cognate NSP oligoduplex (B). Concentration of BsaWI in the samples is increasing from 0.02 to 200 nM (see 'Materials and Methods' section), concentration of DNA is 1 nM. 1 indicates free DNA, 2 – specific BsaWI-DNA complex, 3- a nonspecific complex. (C) Specific DNA binding by the BsaWI mutants. Concentration of BsaWI (wt and mutants) is 3 nM, concentration of DNA – 1 nM. (D) DNA binding constants of the wt and mutant BsaWI.

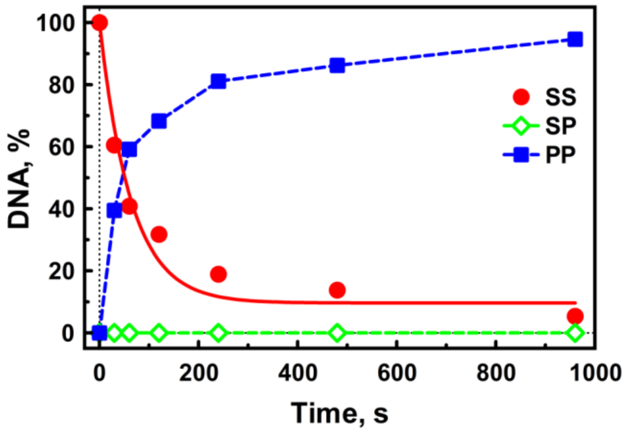

Figure S5. Cleavage of the hairpin substrate HP. The reaction at 37℃ contained 10 nM of the HP DNA (Supplementary Table S1) and 100 nM wt BsaWI in the Reaction buffer (see ‘Materials and Methods’). SS – the substrate, PP – a product cleaved at both strands, SP – HP cleaved at a single strand.

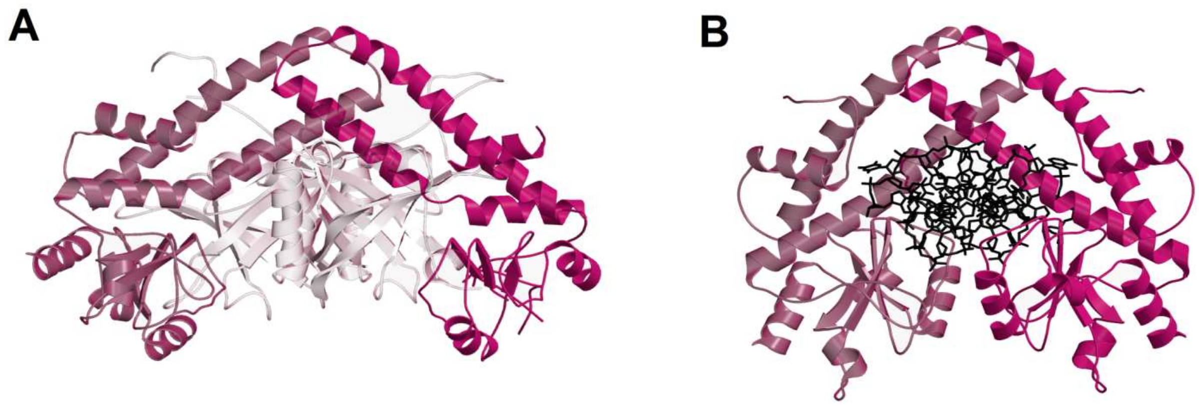

Figure S6. EcoRII-C dimer structure. (A) EcoRII-C dimer (pink) in the apo-structure is open at the catalytic interface; the DNA binding interface is blocked by the N-terminal effector domains (transparent), PDB ID 1NA6 (13). (B) DNA-bound EcoRII-C, PDB ID 3HQG (9).

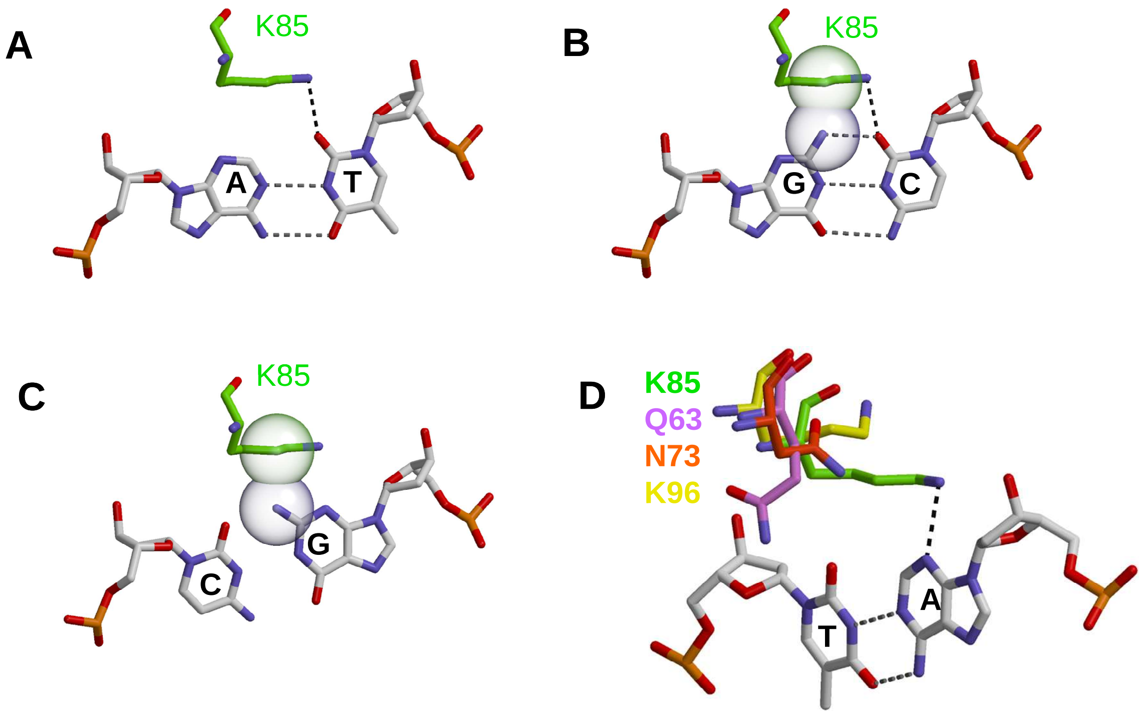

E

Restriction endonuclease	Minor groove contacts	Major groove contacts
Bse634I	N_{73}	$R_{202}P_{203}D_{204}R_{205}$
NgoMIV	Q_{63}	$R_{191}S_{192}D_{193}R_{194}$
SgrAI	K_{96}	$R_{246}S_{247}D_{248}R_{249}$
BsaWI	K_{85}	R_{169} - $D_{170}R_{171}$

Figure S7. Contacts with the outer base pair in the minor groove. (A-C) *In silico* models of K85 with the cognate T:A (A), and the non-cognate C:G (B) and G:C (C) base pairs. (D) Structural overlay of the residues of the CCGG-family REases that make contacts with the outer base pair in the minor groove: Q63 (violet, NgoMIV, 4ABT), N73 (orange, Bse634I, 3V21), K96 (yellow, SgrAI, 3DVO) and K85 (green, BsaWI). DNA is from the BsaWI-DNA complex. (E) Conserved residues of Bse634I/NgoMIV/SgrAI/BsaWI involved in the target recognition. Residues, making contacts with the outer base pairs are depicted in magenta.

SUPPLEMENTARY REFERENCES

50. Sokolowska, M., Kaus-Drobek, M., Czapinska, H., Tamulaitis, G., Szczepanowski, R.H., Urbanke, C., Siksnys, V. and Bochtler, M. (2007) Monomeric restriction endonuclease BcnI in the apo form and in an asymmetric complex with target DNA. J. Mol. Biol., 369, 722-734.
51. Sasnauskas, G., Kostiuk, G., Tamulaitis, G. and Siksnys, V. (2011) Target site cleavage by the monomeric restriction enzyme BcnI requires translocation to a random DNA sequence and a switch in enzyme orientation. Nucleic Acids Res., 39, 8844-8856.
52. Yang, Z., Horton, J.R., Maunus, R., Wilson, G.G., Roberts, R.J. and Cheng, X. (2005) Structure of HinP1I endonuclease reveals a striking similarity to the monomeric restriction enzyme MspI. Nucleic Acids Res., 33, 1892-1901.
53. Horton, J.R., Zhang, X., Maunus, R., Yang, Z., Wilson, G.G., Roberts, R.J. and Cheng, X. (2006) DNA nicking by HinP1I endonuclease: bending, base flipping and minor groove expansion. Nucleic Acids Res., 34, 939-948.
54. Little, E.J., Dunten, P.W., Bitinaite, J. and Horton, N.C. (2011) New clues in the allosteric activation of DNA cleavage by SgrAI: structures of SgrAI bound to cleaved primary-site DNA and uncleaved secondary-site DNA. Acta Crystallogr. D Biol. Crystallogr., 67, 67-74.
55. Skirgaila, R., Grazulis, S., Bozic, D., Huber, R. and Siksnys, V. (1998) Structure-based redesign of the catalytic/metal binding site of Cfr10I restriction endonuclease reveals importance of spatial rather than sequence conservation of active centre residues. J. Mol. Biol., 279, 473-481.
